# Fine-Needle Aspiration Cytology of Parathyroid Carcinoma Mimic Hürthle Cell Thyroid Neoplasm

**DOI:** 10.1155/2014/680876

**Published:** 2014-08-10

**Authors:** Chutintorn Sriphrapradang, Pattana Sornmayura, Niramol Chanplakorn, Objoon Trachoo, Pattarana Sae-Chew, Rangsima Aroonroch

**Affiliations:** ^1^Department of Medicine, Faculty of Medicine, Ramathibodi Hospital, Mahidol University, 270 Rama 6 Road, Rajthevi, Bangkok 10400, Thailand; ^2^Department of Pathology, Faculty of Medicine, Ramathibodi Hospital, Mahidol University, 270 Rama 6 Road, Rajthevi, Bangkok 10400, Thailand; ^3^Research Center, Faculty of Medicine, Ramathibodi Hospital, Mahidol University, 270 Rama 6 Road, Rajthevi, Bangkok 10400, Thailand

## Abstract

*Background*. Fine-needle aspiration (FNA) can cause misdiagnosis of cytomorphological findings between parathyroid and thyroid lesions. *Case Presentation*. A 31-year-old man presented with a palpable neck mass on the right thyroid lobe. FNA cytology was reported as intrathyroidal lymphoid hyperplasia. After 5 years, repeated FNA was done on the enlarged nodule with result of Hürthle cell lesion. Prior to right lobectomy, laboratories revealed elevated serum calcium and parathyroid hormone (PTH). Careful history taking revealed chronic knee pain and ossifying fibroma at the maxilla. Ultrasonography showed a 2.8 cm mass inferior to right thyroid lobe. Pathology from *en bloc* resection was parathyroid carcinoma and immunohistochemical study revealed positivity for PTH. Genetic analysis found somatic mutation of *CDC73* gene in exon1 (c.70delG) which caused premature stop codon in amino acid 26 (p.Glu24Lysfs*2). The final diagnosis was hyperparathyroidism-jaw tumor syndrome. *Conclusions*. FNA cytology of parathyroid can mimic thyroid lesion. It is important to consider and correlate the entire information from clinical history, laboratory, imaging, and FNA.

## 1. Introduction

Parathyroid carcinoma is a rare malignancy, affecting less than 1% of patients with primary hyperparathyroidism [1, 2]. It is usually associated with severe clinical presentations with markedly elevated levels of serum calcium >14 mg/dL and parathyroid hormone (PTH) levels from >5 to 10 times the upper normal limit [3]. Therefore, almost all the patients are symptomatic at the time of presentation [4]. However, it can be an indolent tumor with subtle manifestation reported in 2–7% and up to 30% in the previous case series [5–7]. Patients with parathyroid carcinoma frequently present with a palpable neck mass that can masquerade as a thyroid nodule because of the close localization [8]. The fine-needle aspiration (FNA) can cause misdiagnosis of cytomorphological findings because of significant overlap in cytological features of parathyroid and thyroid lesions. Oncocytic parathyroid adenoma can be confused with Hürthle cell thyroid neoplasm, especially in the absence of clinical information [9].

A high prevalence of parathyroid carcinoma has been demonstrated in patients with hyperparathyroidism-jaw tumor syndrome (HPT-JT; OMIM#145001). It is an exceedingly rare cancer syndrome characterized by primary hyperparathyroidism, ossifying fibromas of the maxilla and mandible, and less commonly renal hamartoma, Wilms' tumor, polycystic kidney disease, degenerative cysts, and/or uterine tumors. Primary hyperparathyroidism is usually the presenting manifestation occurring in late adolescence or in young adulthood. Approximately 15% of these cases have parathyroid carcinoma. A germline inactivating mutation of the* CDC73* tumor suppressor gene (formerly known as* HRPT2* gene; OMIM#607393) can be identified in most patients with HPT-JT and in approximately 20% of patients with sporadic parathyroid carcinoma [10–12]. Also, somatic mutation of* CDC73* gene is present in 60–100% of sporadic parathyroid carcinoma [10, 12–14].

We herein reported a case of a parathyroid carcinoma misinterpreted as Hürthle cell thyroid neoplasm on FNA results and subsequently uncovered HPT-JT.

## 2. Case Report

**Figure 1:**
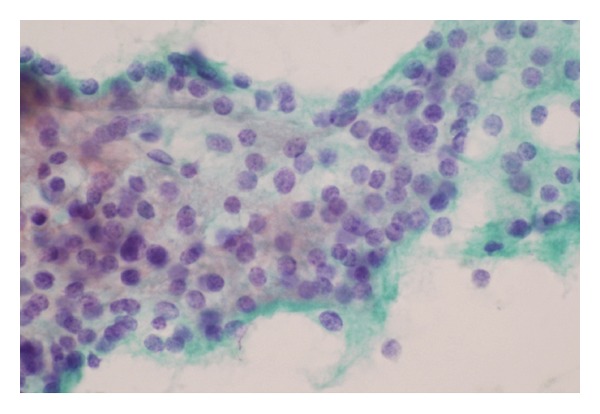
FNA initially reported sheets of follicular cells with oncocytic metaplasia, some naked nuclei, and focal inspissated colloid, compatible with Hürthle cell thyroid lesion. This was difficult to differentiate from parathyroid lesion if lacking of clinical information.

**Figure 2:**
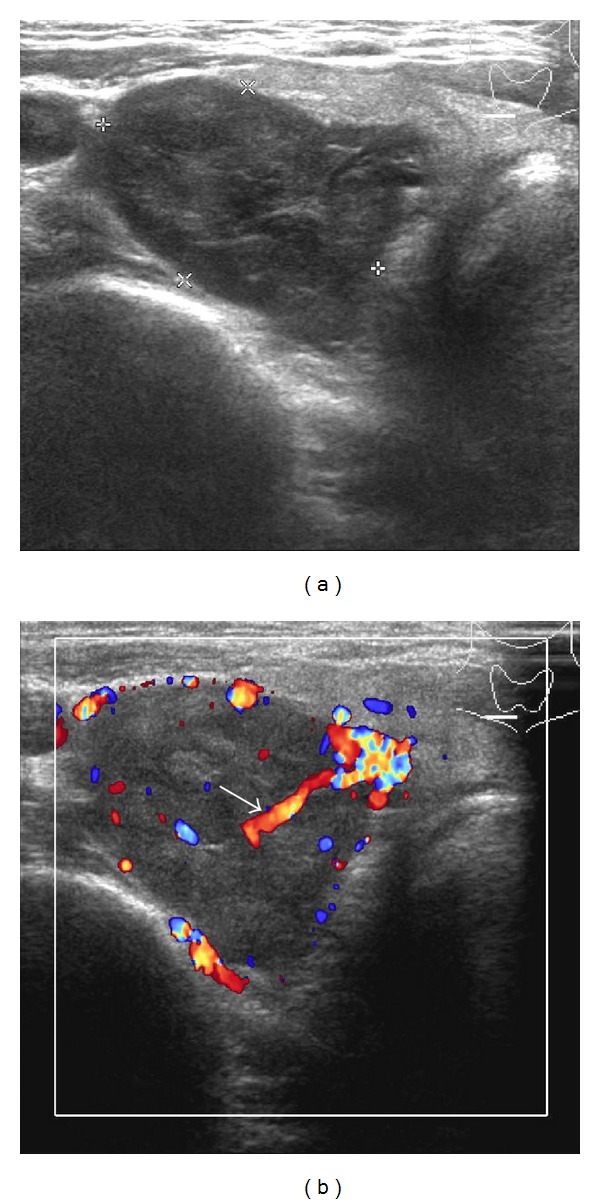
Ultrasonogram of thyroid gland and parathyroid glands. (a) Ultrasonogram and (b) color Doppler flow of right thyroid lobe showed a 2.8 × 1.9 cm heterogenous hypo-to-isoechoic with central hypervascularity solid mass located just inferior to right lobe of thyroid gland. The presence of extrathyroidal feeding artery or polar artery was shown (arrow). This mass was corresponding to palpable neck mass.

**Figure 3:**
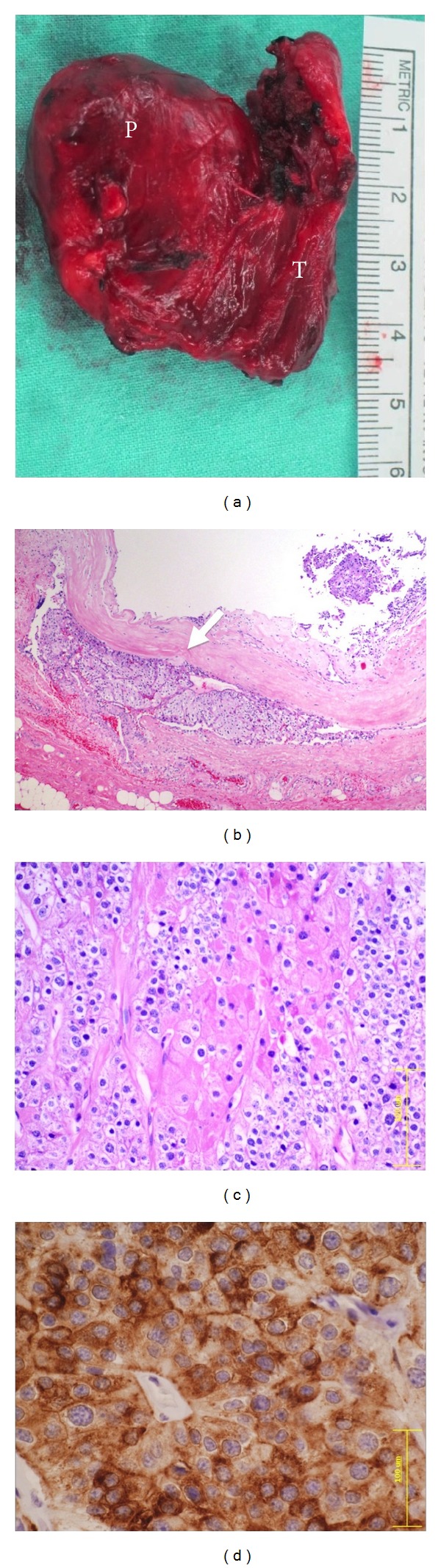
Pathology of parathyroid carcinoma. (a) Gross pathology of parathyroid carcinoma. A large solid tan mass (left, P) was adhered to the right lobe of thyroid gland (right, T). (b) Histopathology demonstrated vascular invasion (arrow) in the tumor capsule (H&E, 40x). (c) The tumor composed mixture of chief cells and oncocytic cells types arranged in solid sheets trabecular and nest pattern (H&E, 200x). (d) Immunohistochemical study for parathyroid hormone (PTH) revealed immunoreactivity in the tumor cells (PTH, 400x).

A 31-year-old man presented with palpable mass on the right side of his neck. Palpation of the thyroid glands showed a 1.5 cm nodule at the lower pole of the right lobe, with no cervical lymphadenopathy. Thyroid function tests were normal. FNA without ultrasound guidance was successfully performed. The cytology was reported as intrathyroidal lymphoid hyperplasia. After observation for 5 years, the size of nodule was slowly enlarged without compressive symptoms. Repeated FNA was done with the result of Hürthle cell lesion (Figure 1). The surgeon decided to perform a right lobectomy. His preoperative laboratory investigations revealed normal thyroid function tests. Serum calcium, phosphorus, and parathyroid hormone (PTH) were 13.5 mg/dL (reference range: 8.5–10.1 mg/dL), 1.9 mg/dL (reference range: 2.5–4.5 mg/dL), and 1,859 pg/mL (reference range: 15–65 pg/mL), respectively. Renal functions were within normal limits. Then, the patient was referred to endocrinologists. On ultrasound examination, a 2.8 cm heterogenous hypervascular mass was located posterior to the right inferior pole of thyroid gland (Figure 2). The technetium-99 m sestamibi single-photon emission computed tomography (Tc-99 m MIBI SPECT) showed increased uptake corresponding to ultrasound findings. Careful systemic history taking revealed that he had been suffering from chronic knee pain for 7 years. Bone radiography showed generalized demineralization, multiple osteolytic lesions at distal femur, proximal tibia, fibula, and patella. Subperiosteal bone resorption along the radial aspect of the middle phalanges and acroosteolysis of digital tufts was shown in X-ray of hands. These radiographic findings were compatible with osteitis fibrosa cystica. Bone mineral density *Z*-scores of lumbar spine, neck of femur, and one-third of distal radius were −3.8, −3.7, and −7.5, respectively, indicating low bone density. Therefore, parathyroid carcinoma was suspected, and* en bloc* resection was performed (Figure 3(a)). After the operation, the patient developed hungry bone syndrome. The pathology result was parathyroid carcinoma which microscopically demonstrated vascular invasion in the tumor capsule (Figure 3(b)). The tumor composed mixture of chief cell and oncocytic cell types with occasional nuclear atypia and prominent nucleoli, arranged in solid sheets trabecular and nest pattern (Figure 3(c)). Immunohistochemical (IHC) study revealed positive immunoreactivity for chromogranin A and parathyroid hormone (Figure 3(d)) but negative immunoreactivity for synaptophysin, calcitonin, and thyroglobulin. This confirmed the parathyroid nature of the lesion.

Additional review of the patient's history revealed the past history of operation for the tumor of the maxilla when he was 10 years old. Pathology showed ossifying fibroma at the right maxilla. Additional computed tomography of chest and abdomen found normal kidney structure and no evidence of metastasis. After informed consent was obtained, we directly sequenced the full coding and flanking splice-junctional regions of the* CDC73* gene in the patient's blood and parathyroid tumor and found a somatic frameshift mutation of* CDC73* gene in exon1 (c.70delG) which caused premature stop codon in amino acid 26 (p.Glu24Lysfs*2). His mother who had history of endometrial cancer of uterus underwent genetic testing for a* CDC73* mutation and was found to be negative. This study was performed with approval from the Ramathibodi Hospital Institutional Review Board. The final diagnosis was parathyroid carcinoma with HPT-JT.

## 3. Discussion

FNA cytology of the parathyroid might be misdiagnosed as Hürthle cell associated lesions of thyroid such as Hürthle cell thyroid neoplasm [9, 15–17]; adenomatous thyroid nodules with Hürthle cell change; or chronic lymphocytic thyroiditis [18] because they might share some cytomorphologic similarities such as follicular structures, colloid-like material in the background [9]. Moreover, the presence of oncocytic cells and naked nuclei of chief cells in parathyroid cytologic specimen can be mimicking Hürthle cells and lymphocytes, respectively [19, 20]. This can be more challenging when the parathyroid lesion is mainly composed of oncocytic cells.

There are some cytomorphologic features that are helpful to differentiate between oncocytic parathyroid and Hürthle cell thyroid neoplasm. Parathyroid cells are smaller and have pale scant cytoplasm in combination with the highly eosinophilic cytoplasm. The cell borders are poorly defined. The nuclei of the parathyroid cells are frequently round to oval with stippled nuclear chromatin or salt-and-pepper appearance and sometimes contain nucleoli. Numerous naked nuclei, approximately the size of erythrocytes, in the background of the smear favor a parathyroid lesion. Hürthle cell thyroid neoplasms have much larger and more prominent nucleoli, and the cells tend to be more dyscohesive [16]. There is no single definite diagnostic criterion that helps to differentiate reliably parathyroid lesions from those of the thyroid, but rather a combination of the cytomorphological features should be applied (Table 1) [21].

In addition to cytomorphology, the clinical information of hyperparathyroidism including pathological fractures, joint and bone pain, muscular asthenia, fatigue, nausea, vomiting, loss of appetite, polyuria, polydipsia, nephrolithiasis, constipation, and weight loss is important. Despite this, these presentations are nonspecific and subtle. High-resolution ultrasonography of neck provides a noninvasive technique to facilitate the differential diagnosis of neck mass. American Thyroid Association recommends that a thyroid ultrasonography should be performed on all patients with suspected thyroid nodules [22]. Furthermore, ultrasound may reveal an unsuspected parathyroid lesion. Normal parathyroid glands are seldom seen on ultrasound. Enlarged parathyroids appear as ovoid mass with homogeneously hypoechoic echogenicity in relation to the thyroid gland and mostly locates adjacent the posterior aspect of the thyroid separated by a fibro-fatty capsule seen as a hyperechoic line between the thyroid gland and the parathyroid mass. Intrathyroidal parathyroid adenoma is about 2–3.4% of cases [23]. Doppler images help in distinguishing suspected parathyroid glands from other structures because parathyroid adenomas typically have a peripheral rim of vascularity and asymmetrically increased blood flow compared to the thyroid. Identification of an extrathyroidal artery (polar artery) feeding a parathyroid mass can discriminate parathyroid glands from lymph nodes, which usually have a hilar blood supply [24]. A panel of IHC study for the PTH, thyroglobulin, thyroid transcription factor-1 (TTF-1), and chromogranin and analyses of the PTH level of the washouts of FNA might be helpful [25, 26]. However, false negative case of PTH staining can occur due to poor cellularity of smears or previously stained smears [21].

Cytologic features can be useful in identifying a parathyroid origin of cells, but it is difficult to make a distinction between normal parathyroid gland and hyperplasia, adenoma, or carcinoma [27]. The appropriate treatment of parathyroid carcinoma is* en bloc* resection which includes resection of the ipsilateral thyroid lobe, together with the isthmus and lymphadenopathy of central compartment of the neck. Thus, it is important to preoperatively identify patients with parathyroid carcinoma to raise the chance of cure by performing complete surgical resection at the initial operation. Several preoperative factors can predict parathyroid carcinoma such as tumor size larger than 3 cm, alkaline phosphatase more than 285 IU/L [28], younger age, hard consistency, high serum calcium, and PTH levels [8, 29].

In addition to thyroid follicular cells, medullary thyroid carcinoma is also included in differential diagnosis for parathyroid cells [30]. Medullary thyroid carcinoma and parathyroid lesions have shared the same features on stippled nuclear chromatin, round to oval cells, loose clusters, and single cells. But the concurrent presence of spindle and oval cells with a granular cytoplasm and an absence of naked nuclei usually are usually considered a diagnosis of medullary thyroid carcinoma. Use of IHC studies for calcitonin in difficult cases has diagnostic value in the differentiation between medullary thyroid carcinoma and parathyroid lesions.

This patient presented with parathyroid carcinoma and later was found to have a history of ossifying fibroma of the maxilla that is compatible with rare endocrine syndrome, HPT-JT. These ossifying tumors are histologically different from the osteitis fibrosa cystica seen in primary hyperparathyroidism. Less commonly (15% of cases), patients suffer from renal complications, for example, various cystic and neoplastic renal abnormalities [31]. Moreover, up to 75% of women with HPT-JT present with uterine tumors [32]. It is an autosomal dominant familial cancer syndrome with variable expression and incomplete penetrance, with 10% of gene carriers showing no clinical manifestations [31]. In the majority of cases, primary hyperparathyroidism is the presenting symptom. A majority of cases with HPT-JT have a single-gland parathyroid involvement and a relatively high risk of parathyroid carcinoma up to 15% [33]. Untreated, HPT-JT may result in significant morbidity and mortality due to severe hypercalcemia. The* HRPT2* gene, also known as* CDC73*, is located at chromosome 1q31.2 underlining the HPT-JT syndrome [11].* CDC73* consists of 17 exons and encodes 531 amino acids. It codes for a protein known as parafibromin that acts as a tumor suppressor involving the regulation of transcriptional events and histone modification; however, the exact mechanism by which parafibromin serves as a tumor suppressor remains unknown. Mutations in* CDC73* are scattered throughout the coding region and splice sites and most mutations are predicted to cause inactivation of the protein product. About 80% of germline mutations have been reported in exons 1, 2, and 7 [34]. The majority of mutations are frameshift mutations, and a smaller proportion of mutations are nonsense and missense. Most mutations (more than 80%) result in premature truncation of parafibromin. Additionally, some patients with whole gene deletions have also been reported [35]. Somatic mutation of* CDC73* is uncommon in sporadic benign parathyroid adenomas [36]. In contrast, mutations of* CDC73* are frequently seen in apparently sporadic cases of parathyroid carcinoma [10, 12]. Our patient was found to have a frameshift somatic mutation in exon 1 causing premature stop codon. This mutation has been reported in ossifying fibroma of the jaw [37]. The identification of a mutation in the coding region would provide a definitive diagnosis, although a negative result could not exclude the possibility of a mutation in the promoter or other noncoding regions of* CDC73*. Carpten et al. reported that* CDC73* sequencing fails to identify a germline mutation in about half of the index patients from families with classic HPT-JT and with proven genetic linkage to 1q24-q32 [11]. Unidentified germline mutation may locate in the 5′-regulatory region of* CDC73.* Gross deletions or rearrangements in* CDC73* may not be readily identified using direct sequencing. Also it is possible that other tumor suppressor genes may contribute to the development of parathyroid carcinoma.

Accordingly, all patients with newly diagnosed parathyroid carcinoma should have a meticulous review of family history and should be considered for* CDC73* mutation screening whether or not other features of HPT-JT are present. Genetic tests of this recognized mutation in at-risk asymptomatic family members would emphasize regular clinical and biochemical surveillance of carriers of the mutation and reassure to family members who have no mutation. Children with the gene mutation are recommended to undergo baseline screening of calcium and PTH levels, jaw X-rays, and renal ultrasound as early as age 5 [38]. Monitoring serum calcium concentrations in all subjects at risk provides an alternative to definitive genetic diagnosis.

## 4. Conclusions 

It is difficult to distinguish parathyroid lesions from thyroid lesions based on solely FNA cytomorphologic diagnosis because of their morphologic similarities. Therefore, it is important to consider and relate the whole information from careful clinical history taking, laboratory, imaging studies, and FNA. PTH assay in FNA specimen and immunohistochemical study can definitely distinguish between parathyroid and thyroid lesions. HPT-JT is a rare condition that physicians should recognize and genetically counsel the patient and family members for regular surveillance for early detection of complications.

## Figures and Tables

**Table 1:** Comparison features between oncocytic parathyroid lesions and Hürthle cell thyroid neoplasm.

Cytomorphologic features	Oncocytic parathyroid lesions	Hürthle cell thyroid neoplasm
Patterns	Cells isolated and in loose aggregates or syncytial fragments; nuclear overlapping molding; and anisokaryosis	Cells in syncytial fragments; more dyscohesive

Cells	Small 6–9 *μ*m in diameter; round to cuboidal	Larger than parathyroid; variable in size

Naked nuclei	Numerous	Few

Nuclei	Small; coarsely granular chromatin	Variably enlarged; fine to coarsely chromatin

Nucleoli	Inconspicuous	Prominent

Cytoplasm	Scant; clear, granular, or eosinophilic	Scant

Colloid	Absent but colloid-like material	Scant/−

IHC studies		
PTH	+	−
TTF-1	−	+
Thyroglobulin	−	+
Chromogranin	+	−

PTH: parathyroid hormone; TTF-1: thyroid transcription factor-1; IHC: immunohistochemical.
